# Synergism between *Streptomyces viridosporus* HH1 and *Rhizophagus irregularis* Effectively Induces Defense Responses to Fusarium Wilt of Pea and Improves Plant Growth and Yield

**DOI:** 10.3390/jof8070683

**Published:** 2022-06-28

**Authors:** Hany H. A. El-Sharkawy, Younes M. Rashad, Nahla T. Elazab

**Affiliations:** 1 Mycology Research and Plant Disease Survey Department, Agricultural Research Center, Plant Pathology Research Institute, Giza 12211, Egypt; drhanylasheen@arc.sci.eg; 2 Plant Protection and Biomolecular Diagnosis Department, Arid Lands Cultivation Research Institute, City of Scientific Research and Technological Applications (SRTA-City), New Borg El-Arab City 21934, Egypt; 3 Botany Department, Faculty of Science, Mansoura University, Mansoura 35516, Egypt; nahlatharwat83@gmail.com

**Keywords:** colonization, resistance, *Fusarium oxysporum*, *Pisum sativum*, mycorrhizal

## Abstract

Fusarium wilt is a detrimental disease of pea crop, resulting in severe damage and a reduction in its yield. Developing synergistically enhanced bioagents for disease management and growth promotion is pivotal for food safety, security, and sustainability. In this study, biocontrol potential of treating pea plants with *Streptomyces* *viridosporus* HH1 and/or their colonization with *Rhizophagus* *irregularis* against infection with Fusarium wilt was investigated. Impacts on the expression profiles of defense-related genes, biochemical, and ultrastructural levels, as well as the growth and yield of pea plants in response to these treatments, were also investigated. Data obtained indicated the antifungal activity of *S. viridosporus* HH1 against *F. oxysporum* f.sp. *pisi* in vitro. Furthermore, the GC-MS analysis revealed production of different bioactive compounds by *S. viridosporus* HH1, including 2,3-butanediol, thioglycolic acid, and phthalic acid. The results from the greenhouse experiment exhibited a synergistic biocontrol activity, resulting in a 77% reduction in disease severity in pea plants treated with *S. viridosporus* HH1 and colonized with *R. irregularis.* In this regard, this dual treatment overexpressed the responsive factor *JERF3* (5.6-fold) and the defense-related genes *β*-1,3-glucanase (8.2-fold) and the pathogenesis-related protein 1 (14.5-fold), enhanced the total phenolic content (99.5%), induced the antioxidant activity of peroxidase (64.3%) and polyphenol oxidase (31.6%) enzymes in pea plants, reduced the antioxidant stress, and improved their hypersensitivity at the ultrastructural level in response to the Fusarium wilt pathogen. Moreover, a synergistic growth-promoting effect was also recorded in pea plants in response to this dual treatment. In this regard, due to this dual treatment, elevated levels of photosynthetic pigments and improved growth parameters were observed in pea leaves, leading to an increment in the yield (113%). In addition, application of *S. viridosporus* enhanced the colonization levels with *R. irregularis* in pea roots. Based on the obtained data, we can conclude that treating pea plants with *S. viridosporus* HH1 and colonization with *R. irregularis* have synergistic biocontrol activity and growth-promoting effects on pea plants against Fusarium wilt. Despite its eco-safety and effectiveness, a field evaluation of this treatment before a use recommendation is quite necessary.

## 1. Introduction

Common pea (*Pisum sativum* L.) is one of the widely cultivated vegetable legumes worldwide, ranking third after soybean and dry bean, with a total production of 19,866,601 tons in 2020 [1]. This popular crop is grown for its fresh and dry seeds, green pods, and foliage, which are used for food and feed. It is nutritious due to its high contents of protein (23–31%), minerals, carbohydrates, and fiber [2]. However, the pea is susceptible to an array of fungal and bacterial diseases, which may cause severe damage to pea production.

Fusarium wilt, caused by *Fusarium oxysporum* f.sp. *pisi* W.C. Snyder and H.N. Hansen is a detrimental disease which infects pea crops, causing severe damage up to a full yield loss under favorable conditions. *Fusarium oxysporum* is a soil-borne pathogen and can be disseminated to new uninfected areas via agricultural tools, seeds, wind, or water. A large number of microconidia, macroconidia, and chlamydospores are produced by the pathogen, which can survive in the soil for more than ten years [3]. The spore germinates in soil, penetrates the plant roots, and extends in growing upward in the stem through the xylem vessels, blocking the water passage and causing plant wilt. Pea can be infected with Fusarium wilt at any growth stage. The disease symptoms include leaves downward curling, yellowing, plant stunting, stem thickening at the soil line, xylem vessels discoloration (brown to black), and finally, complete death. The infection appears in circular to oval patterns in the field [4]. The control of pea Fusarium wilt has been reported using many chemical fungicides, including thiophanate-methyl, metalaxyl, dimethomorph, and mancozeb [5]. However, the use of chemical fungicides is unfavorable due to their hazardous health effects on humans and animals, and soil microbiome diversity [6]. In recent decades, the biological control of plant diseases has emerged as a promising and safe alternative to chemical fungicides. In this regard, the biocontrol activity of different fungal and bacterial bioagents has been studied against various fungal plant diseases [7]. 

*Streptomyces* spp. are Gram-positive, spore-producing, filamentous bacteria and represent the richest source for antifungal, antibacterial, and antiviral secondary metabolites [8]. More than 70% of the antibiotics discovered so far have been sourced from members belonging to this genus [9]. In addition, a wide array of bioactive secondary metabolites, including siderophores, phytohormones, volatile organic compounds, and enzymes, is produced by *Streptomyces* spp. [10]. Production of a diverse set of bioactive metabolites by *Streptomyces* spp. Makes them promising candidates as biocontrol agents against different phytopathogens [11]. The biological control of fungal plant diseases using *Streptomyces* spp. Has been extensively studied in recent decades [12,13,14,15]. In this context, Rashad et al. [16] reported a considerable reduction in the disease severity of tomato plants infected with Fusarium wilt disease when treated with the bioagent *S. griseorubens* E44G. Moreover, a significant enhancement in the growth and yield of tomato plants was also reported. The efficient biocontrol potential was attributed to the production of the antifungal chitinase enzyme. Different antagonistic mechanisms have been reported for *Streptomyces* spp. [17,18]. However, these modes of action depend on many conditions, such as the plant species, the target pathogen, soil conditions, and the surrounding microorganisms [19]. In addition, some *Streptomyces* strains can colonize the plant tissues and serve as plant growth promoters via nutrient acquisition or the production of phytohormones [20]. 

Among the bioagents widely studied for their biocontrol activity against different plant diseases is the arbuscular mycorrhizal fungi (AMF) [7,21,22,23]. They are biotrophic fungi belonging to the subphylum Glomeromycotina, which colonizes the roots of around 80% of terrestrial plants, forming a mutualistic association [24]. In this symbiotic relationship, the fungus partner benefits from the carbohydrates formed by the host via photosynthesis. Additionally, the host plant receives multiple benefits from the fungus partner, including the promotion of the plant’s growth [25], water and nutrient acquisition [26], the induction of the plant’s resistance against many abiotic stresses, such as salinity and drought [27], and by triggering plant immunity against various plant diseases [28]. Rashad et al. [29] reported a significant overexpression in many polyphenol biosynthetic pathway genes in mycorrhizal sunflower plants colonized with *Rhizophagus irregularis* and infected with Rhizoctonia root rot. In addition, the disease severity was considerably reduced, and the plant growth was promoted due to the mycorrhization, compared with the non-mycorrhizal plants.

Different immunity responses have been reported to be activated against invading phytopathogens via multiple signaling pathways, which interact with each other. Jasmonate and ethylene-responsive factor 3 (*JERF3*) has a pivotal defensive function in regulating a set of defense-related genes via both the jasmonate and ethylene pathways [27]. One of the most important antifungal genes is *β*-1,3-glucanase (*GLU*), which has a degrading function on the *β*-1,3/1,6-glucosidic bonds in the glucan molecule of the fungal cell wall [30]. In addition, the pathogenesis-related (PR) gene (*PR1*) is another antifungal gene, which is involved in the plant’s defense responses to various fungal pathogens [31]. This work aimed to (1) investigate the antifungal activity of *S. viridosporus* HH1 against *F. oxysporum* f.sp. *pisi* in vitro; (2) evaluate the biocontrol activity by treating pea seeds with *S. viridosporus* HH1 and/or the colonization of their roots with *R. irregularis* against Fusarium wilt within the greenhouse; (3) study the impacts of these treatments on the transcriptional expression of the responsive factor *JERF3* and the two defense-related genes; (4) investigate their effects on the ultrastructure of pea root; and (5) evaluate their effects on the plant growth and biochemical responses.

## 2. Materials and Methods

### 2.1. Pea Cultivar, Pathogen, and Bioagents

Pea seeds cv. Master-B were kindly provided by the Horticultural Research Institute, Agricultural Research Center, Giza, Egypt and utilized during the greenhouse evaluation. 

The fungal pathogen was isolated from diseased pea plants, collected from pea fields in the Al-Sharkia governorate, showing typical wilt symptoms. The isolated causal agent was purified using a single spore technique and was then morphologically identified as *F. oxysporum* f.sp. *pisi* according to its cultural and microscopic characteristics, according to Summerell et al. [32] and Booth [33]. Koch’s postulates were fundamentally fulfilled to ensure that this fungus was the causal organism. The identification of the pathogenic isolate was carried out at the Plant Pathology Research Institute, Agricultural Research Centre, Egypt, and the isolate was then deposited in the culture collection at the Plant Pathology Research Institute Fungarium. Inoculum of the pathogen was prepared by the cultivation of the pathogenic fungus on sterilized soil: sorghum (2:1 *v*/*v*) medium and incubated at 25 ± 2 °C for 10 days. 

An active strain of *S. viridosporus* HH1 was kindly obtained from the Bacterial Diseases Research Department, Plant Pathology Research Institute, Agricultural Research Centre, Egypt. For the inoculum preparation, *S. viridosporus* HH1 was cultivated in a potato dextrose broth for 7 days at 30 °C. A spore suspension was prepared and adjusted at 10^6^ spore mL^−1^.

The mycorrhizal fungus *R. irregularis* (Blaszk., Wubet, Renker & Buscot) Walker & Schüßler was obtained from the Agricultural Research Centre, Egypt. For the preparation of the mycorrhizal inoculum, monosporic cultures (80% colonization index) of *R. irregularis*, propagated using sudangrass as the host plants, were used. The inoculum was composed of the fungal spore, rhizospheric soil, and mycorrhizal roots’ pieces.

### 2.2. Assessment of Antifungal Activity of S. viridosporus HH1 In Vitro 

Using the dual culture plate technique, the antifungal activity of *S. viridosporus* HH1 was assessed against *F. oxysporum* f.sp. *pisi*. On a potato dextrose agar (PDA, Difco, Detroit, MI, USA) plate, a 7 mm disc, was taken from a week-old culture of *F. oxysporum* f.sp. *pisi* and was inoculated 3 cm from the plate’s edge. At 2 cm from the opposite edge of the plate, a loop of *S. viridosporus* HH1 was streaked. For the control treatment, the PDA plates were inoculated only with the fungal pathogen. Five replicates were used for each treatment. All plates were incubated at 28 ± 2 °C until the control plates were fully covered by the pathogen. The inhibition in the mycelial growth of the fungus was measured compared to that in the control plate using the following equation:

(1) $$\mathrm{Growth}\;\mathrm{Inhibition}\;\left(\%\right)=\frac{R\;1\;-\;R\;2}{R\;1}\times 100$$

where *R*1 = the inward linear growth in the control plate and *R*2 = the inward linear growth in the dual culture plate.

### 2.3. Gas Chromatography/Mass Spectrometry (GC/MS) Analysis

For the identification of the secondary metabolites produced by *S. viridosporus* HH1, the composition of its culture filtrate was analyzed using a GCMS-QP2010 system (Shimadzu, Kyoto, Japan) equipped with a mass selective detector (MS). A culture filtrate of *S. viridosporus* HH1, cultivated in a potato dextrose broth for 7 days at 30 °C, was used in this analysis. The analysis was carried out under the following conditions: detector mass spectrometer voltage of 75 eV at a max temperature of 250 °C. The capillary column (TRB-5MS, 30 m × 0.25 mm × 0.25 µm) was used with helium as a carrier gas. The analysis was started at 50 °C for 1 min, and the oven temperature was increased at 15 °C min^−1^ until 180 °C, where it was held for 1 min before it was increased again until 230 °C at 7 °C min^−1^, held for 2 min, and finally increased to 250 °C at 10 °C min^−1^. The identity of the ingredients was determined by comparing their retention time and mass spectrum with the database of the National Institute of Standards and Technology (NIST 11) Spectral Library (Gaithersburg, MD, USA).

### 2.4. Greenhouse Evaluation

Pots (30 cm diameter) full of disinfected soil and a mixture of clay and sand (1:1, *v*/*v*) were used. Surface sterilized pea seeds, using sodium hypochlorite (5%), were planted at 10 seeds per pot. Half of the pots were inoculated with the *R. irregularis* inoculum under each seed at 10 g seed^−1^. For the soil infestation, the upper layer of the soil was mixed with the inoculum of *F. oxysporum* f.sp. *pisi*. at 3% (*w*/*w*) and watered 10 days before planting. For the application of *S. viridosporus* HH1, the pea seeds were mixed before planting with a freshly prepared spore suspension amended with gum arabic (1%). The pea seeds were soaked for 3 h in the chemical fungicide Tendro 40% FS (carboxin 20% + thiram 20%) at 3.5 mL/kg of seeds before the planting served as a positive control. Another set of pots that did not receive any treatments was used as a negative control. Each treatment was replicated five times (pots). All pots were irrigated as necessary and did not receive any fertilization. The applied treatments were as follows: CNM: uninfected, untreated, and non-mycorrhizal, M: uninfected, untreated, and mycorrhizal, PNM: infected, untreated, and non-mycorrhizal, PM: infected, untreated, and mycorrhizal, SNM: uninfected, treated with *S. viridosporus* HH1, and non-mycorrhizal, SM: uninfected, treated with *S. viridosporus* HH1, and mycorrhizal, PFNM: infected, treated with chemical fungicide, and non-mycorrhizal, PFM: infected, treated with chemical fungicide, and mycorrhizal, PSNM: infected, treated with *S. viridosporus* HH1, and non-mycorrhizal, PSM: infected, treated with *S. viridosporus* HH1, and mycorrhizal. The pots were arranged in a factorial design (split-plot), composed of five treatments (C, P, S, PS and PF) and two levels of mycorrhizal status (M or NM), and kept under greenhouse conditions at 28/18 °C (day/night), and 70% relative humidity.

#### 2.4.1. Gene Expression Profiling 

For the molecular investigation, samples of pea roots were collected 14 days post planting (dpp). From each sample, mRNA was extracted using the RNeasy Mini Kit (Qiagen, Hilden, Germany). The cDNA was synthesized using a SureCycler 8800 (Agilent, Santa Clara, CA, USA). The reaction mixture (20 μL) contained 3.5 μL RNase-free water, 3 μL dNTPs (10 mM), 3 μL 5 × reaction buffer, 7 μL oligo (dT) primer (5 pmol μL^−1^), 3 μL RNA (30 ng) and 0.5 μL reverse transcriptase enzyme (New England Biolabs, Frankfurt, Germany). The reaction was performed as follows: 42 °C for 1 h, and 70 °C for 10 min. 

A quantitative real-time PCR (qPCR) reaction was performed using a Rotor-Gene-6000-system (Qiagen, Germantown, MD, USA). The reaction mixture contained 1.5 μL RNase-free water, 3 μL cDNA, 12.5 μL 2xSYBR^®^ Green RT Mix (Bioloine, Luckenwalde, Germany), and 1.5 μL for each of the forward and reverse primers (10 pmol μL^−1^). The primer sequences of the studied genes are presented in Table 1. The qPCR reaction was performed as follows, one cycle at 95 °C for 3 min, 45 cycles (95 °C for 15 s, 56 °C for 30 s and 72 °C for 30 s). Due to its high stability in the mycorrhizal plants colonized with *R. irregularis*, elongation factor 1 α (EF1 α) was used as a reference gene [34]. The relative expression level was determined using the comparative CT method (2^−∆∆CT^) [35]. Three biological and three technical replicates were applied for each sample.

#### 2.4.2. Growth and Yield Evaluation 

Fifty dpp, five plants of each treatment were carefully uprooted, washed under tap water, and evaluated for their shoot height (cm), root length (cm), shoot and root dry weight (g), number of leaves per plant, and leaf area (cm^2^). Seventy dpp, five plants of each treatment were evaluated for the number of pods per plant, pod weight (g), pod length and width (cm), number of seeds per pod, and yield per plant (g)]. For the weight determination, the samples were first dried in a hot oven at 80 °C for 3 days. 

#### 2.4.3. Disease Assessment

Forty-five dpp, five plants of each treatment were evaluated for the disease severity (DS) of Fusarium wilt using a six-level scale according to Bani et al. [36], where 0 = no symptoms; 1 = chlorosis or wilting of one basal leaf, pale yellow-green, downward curling of leaf margins and stipules; 2 = chlorosis or wilting of some basal leaves, no stunting; 3 = chlorosis or wilting of several basal leaves, slight stunting and yellowing of most leaves; 4 = chlorosis or wilting of most leaves, heavy stunting, and the drying of lower leaves; 5 = death of the seedling. The DS was calculated using the following equation:

(2) $$\mathrm{DS}\;\%=\frac{\Sigma ab}{AK}\times 100$$

where *a* = the number of wilted plants at the same level, *b* = the severity level, *A* = the total number of evaluated plants, and *K* = the highest severity level. 

#### 2.4.4. Phenolic Content and Activity of Antioxidant Enzymes

Thirty dpp, five pea roots of each sample were analyzed for the total phenolic content and enzyme activity of peroxidase (POD) and polyphenol oxidase (PPO). The total phenolic content was determined according to the method of Malik and Singh [37]. For the extraction of the phenolic compounds, the root sample (1 g) was ground in 10 mL of ethanol (80%) and centrifuged at 5000 rpm for 15 min. The supernatant was collected, and the solvent was fully evaporated. Then, the residue was re-dissolved in 5 mL of distilled water. For the estimation, 0.2 mL of the extract was made up to 3 mL with distilled water, and 0.5 mL of Folin–Ciocalteu reagent was added. After 3 min, 2 mL of Na_2_CO_3_ 20% was added to the mixture and kept in boiling water for 1 min. The absorbance was measured at 650 nm against a blank. Three samples were applied for each treatment. 

To prepare the enzyme crude extract, the root sample (1 g) ground in 2 mL of phosphate buffer (0.2 M, pH 7.0) was centrifuged at 5000 rpm for 15 min under cooling. The activity of the POD was estimated according to Maxwell and Bateman [38], while the PPO was estimated according to Galeazzi et al. [39]. Three samples were applied for each treatment. 

#### 2.4.5. Biochemical Analyses

Forty dpp, the pea leaves collected from the same level (third upper leaf) were analyzed for the total photosynthetic pigments according to Harborne [40]. The leaf sample (3 g) was homogenized in 10 mL of acetone (85%) and centrifuged at 5000 rpm for 15 min. The supernatant was made up to a known volume with acetone. The absorbance was measured at 452.5, 644 and 663 nm against a blank. Fifty dpp, pea roots of each treatment were analyzed for electrolyte leakage using an EC Meter (Hana, Leighton Buzzard, UK) to determine the membrane permeability (%), as described by Shi et al. [41]. The total soluble solids (TSS, °Brix) in pea seeds were estimated using a hand refractometer (Master T, ATAGO Co., Minato-ku, Japan). Three samples were applied for each treatment.

#### 2.4.6. Evaluation of Mycorrhization Level

Thirty dpp, the pea roots of each treatment were evaluated for the colonization level with *R. irregularis*. The root segments (1 cm) were boiled in potassium hydroxide (10%) and stained using trypan blue (0.05%) (Sigma, St. Louis, MO, USA), as described by Phillips and Hayman [42]. The colonization level was estimated using a light microscope according to Trouvelot et al. [43]. The root segments were evaluated for their frequency and intensity of colonization, as well as their arbuscules frequency. 

#### 2.4.7. Ultrastructural Investigation

Forty dpp, the samples of pea roots were examined for ultrastructural changes using a transmission electron microscope (TEM) (JEM-1230, JEOL Ltd., Tokyo, Japan). A root segment (1 cm^2^) was gradually treated with ethyl alcohol (10–100%), each for 10 min. The dehydrated sample was treated with ethanol-propylene oxide and propylene oxide–Araldite, embedded in gelatin capsules, and heated at 60 °C for 60 h. Ultrathin sections were prepared using a Reichert Ultramicrotome. The sections were stained with uranyl acetate and then lead citrate before the examination.

### 2.5. Statistical Analyses

Statistical analyses of the obtained results were performed using the CoStat software V 6.4 (Costat Institute, Cary, NC, USA). At first, the results were examined for normality and subjected to an analysis of variance. Mean values were compared using the least significant difference (LSD) or Tukey’s HSD test at a *p* ≤ 0.05 (on a one-way ANOVA). 

## 3. Results

### 3.1. Antifungal Activity of S. viridosporus HH1 against F. oxysporum f.sp. pisi In Vitro

**Figure 1:**
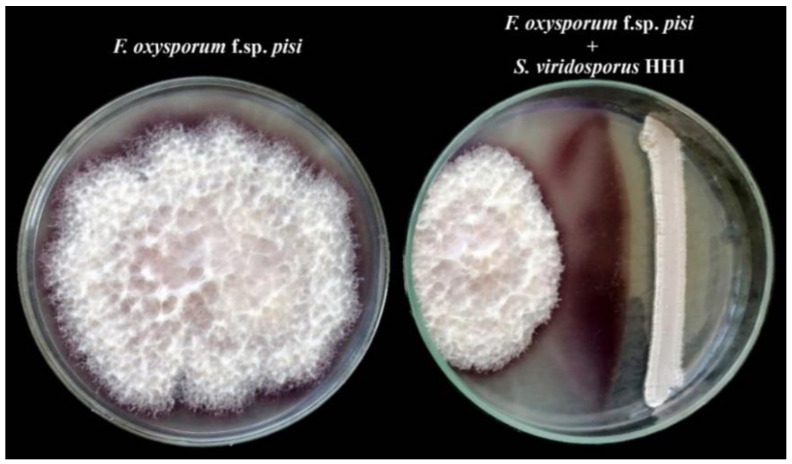
A dual culture test showing the antifungal activity of *S. viridosporus* HH1 against *F. oxysporum* f.sp. *pisi*.

Streptomyces viridosporus HH1 was screened for their antifungal activity against *F. oxysporum* f.sp. *pisi* in vitro. The obtained results showed that *F. oxysporum* f.sp. *pisi* freely grew in the control plate, covering the full plate. In contrast, a considerable inhibition (63%) in the mycelial growth of *F. oxysporum* f.sp. *pisi* was observed in the dual culture plate due to *S. viridosporus* HH1, compared to the control plate (Figure 1). This result indicated the antifungal activity of *S. viridosporus* HH1 against *F. oxysporum* f.sp. *pisi*. 

### 3.2. GC-MS

The secondary metabolites produced by *S. viridosporus* HH1 were identified using the GC-MS system (Table 2). Twenty compounds were detected in varying proportions. The major secondary metabolites included 2,3-butanediol (17.26%), phthalic acid, di(3,5-dimethylphenyl) ester (13.78%), thioglycolic acid (13.35%), tetradecamethyl cycloheptasiloxane (11.74%), 2-ethoxypropane (9.21%), and d-2,3-butanediol (8.9%). In addition, some metabolites were detected at intermediate proportions, including phloroglucinol, tris(trimethylsilyl ether) (6.94%), tetradecamethyl hexasiloxane (6.65%), dodecamethyl cyclohexasiloxane (4.88%), and 1,2-Diphenyltetramethyldisilane (2.33%), while the other metabolites were found in minor ratios.

### 3.3. Gene Expression Profiling

**Figure 2:**
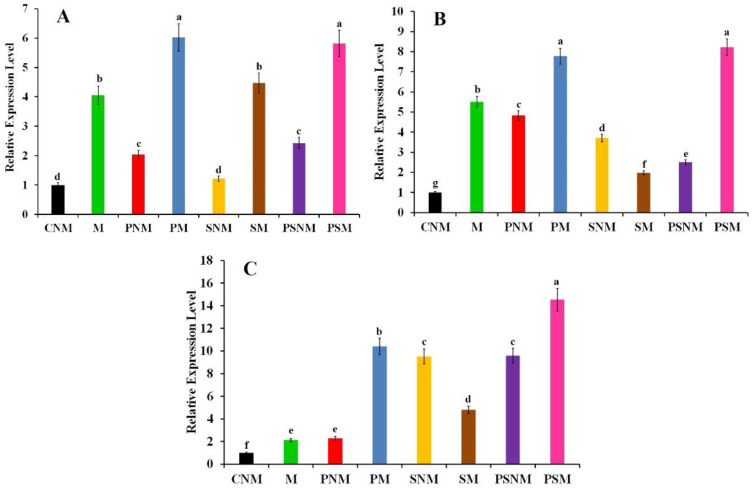
Histograms showing the gene expression profiles of the responsive factor *JERF3* (**A**) and the defense-related genes *GLU* (**B**) and *PR1* (**C**) in pea roots infected with Fusarium wilt in response to application of *S. viridosporus* HH1 and/or colonization with *R. irregularis* two weeks post planting. Where CNM: uninfected, untreated, and non-mycorrhizal, M: uninfected, untreated, and mycorrhizal, PNM: infected, untreated, and non-mycorrhizal, PM: infected, untreated, and mycorrhizal, SNM: uninfected, treated with *S. viridosporus* HH1, and non-mycorrhizal, SM: uninfected, treated with *S. viridosporus* HH1, and mycorrhizal, PSNM: infected, treated with *S. viridosporus* HH1, and non-mycorrhizal, PSM: infected, treated with *S. viridosporus* HH1, and mycorrhizal. For each gene, columns superscripted with the same letter are not significantly different according to Tukey’s HSD test at *p* ≤ 0.05. Three biological and three technical replicates were applied for each treatment. Error bars represent standard errors.

The transcriptional expression level of *JERF3*, the defense-related genes *GLU* and *PR1* in infected pea root in response to the application of *S. viridosporus* HH1 and/or colonization with *R. irregularis*, was quantified via qPCR two weeks post planting (Figure 2). The obtained results revealed that these treatments led to the overexpression of the transcription factor *JERF3* to varying degrees. Compared with the non-mycorrhizal control plants, mycorrhization of the infected pea roots with/without the application of *S. viridosporus* HH1 achieved the highest relative expression levels, recording 6- and 5.8-fold, respectively. The mycorrhization of the uninfected pea roots with/without the application of *S. viridosporus* HH1 came second in this regard. Regarding *GLU*, data from the qPCR showed that all the single or combined treatments significantly upregulated its expression, at varying degrees, compared with the non-mycorrhizal control plants. In this regard, the highest relative expression levels were recorded for the mycorrhizal-infected pea roots treated or not treated with *S. viridosporus* HH1, recording 8.2- and 7.8-fold, respectively. The mycorrhizal-uninfected pea treatment ranked second, recording 5.5-fold when compared to the non-mycorrhizal control plants. For *PR1*, all the tested treatments overexpressed the gene expression compared with the non-mycorrhizal control plants. The highest expression level was recorded for the mycorrhizal-infected pea roots treated with *S. viridosporus* HH1, recording 14.5-fold, followed by the mycorrhizal-infected pea roots, which were recorded 10.4-fold.

### 3.4. Growth Parameters

The effects of the application of *S. viridosporus* HH1 and/or colonization with *R. irregularis* on the growth parameters of pea plants infected with Fusarium wilt at 50 dpp are presented in Table 3. Data obtained from the greenhouse experiment revealed that the infection of pea plants with Fusarium wilt considerably reduced all evaluated growth parameters. Compared with the infected non-mycorrhizal plants, treating pea plants with *S. viridosporus* HH1 and/or colonization with *R. irregularis* significantly enhanced their shoot height, root length, root dry weight, number of leaves per plant, and leaf area, regardless of whether the plants were infected or not. Except for the infected plants treated only with *S. viridosporus* HH1, all tested treatments improved the shoot dry weight of pea plants. Moreover, the application of *S. viridosporus* HH1 and/or colonization with *R. irregularis* improved all evaluated growth parameters in the uninfected pea plants when compared with the non-treated, non-mycorrhizal control plants. Except for the leaf area, treating infected pea plants enhanced the evaluated growth parameters. In general, the colonization of pea plants with *R. irregularis* was more effective than their treatment with *S. viridosporus* HH1 in most of the evaluated parameters. However, the dual treatment mostly recorded the highest values in this context.

### 3.5. Yield and Its Components

The results presented in Table 4 indicate the mean values of yield and its components of pea plants infected with Fusarium wilt and treated with *S. viridosporus* HH1 and/or colonized with *R. irregularis* at 70 dpp. The infection of pea plants with Fusarium wilt considerably reduced all evaluated yield parameters, except the number of pods per plant, compared with the uninfected non-mycorrhizal plants. The application of *S. viridosporus* HH1 and/or colonization with *R. irregularis* led to an enhancement in all yield parameters in infected pea plants when compared to the infected, untreated, and non-mycorrhizal pea plants. In addition, the tested treatments improved the number of pods per plant, pod length, and yield per plant of the uninfected pea plants compared with the uninfected non-mycorrhizal plants. Treating infected pea plants with the chemical fungicide improved the pod weight, length, width, and yield per plant when compared with the untreated infected plants. For yield per plant, the colonization of pea plants with *R. irregularis* was more effective than their treatment with *S. viridosporus* HH1. However, the dual treatment recorded the highest yield value.

### 3.6. Disease Severity

**Figure 3:**
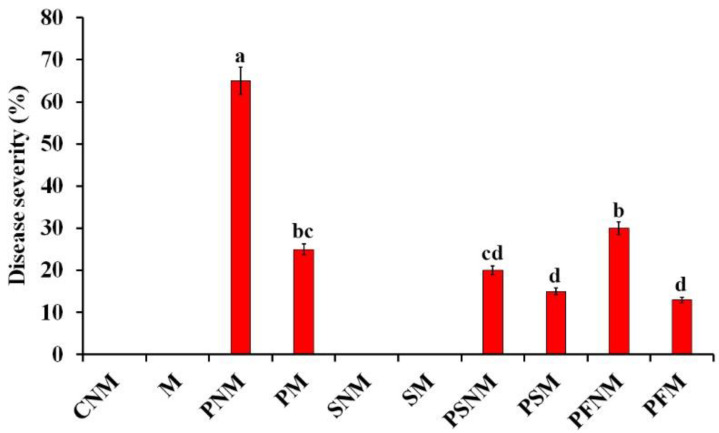
Histogram showing the disease severity of Fusarium wilt in pea plants in response to treatment with *S. viridosporus* HH1 and/or colonization with *R. irregularis* at 40 days post planting. Where CNM: uninfected, untreated, and non-mycorrhizal, M: uninfected, untreated, and mycorrhizal, PNM: infected, untreated, and non-mycorrhizal, PM: infected, untreated, and mycorrhizal, SNM: uninfected, treated with *S. viridosporus* HH1, and non-mycorrhizal, SM: uninfected, treated with *S. viridosporus* HH1, and mycorrhizal, PFNM: infected, treated with chemical fungicide, and non-mycorrhizal, PFM: infected, treated with chemical fungicide, and mycorrhizal, PSNM: infected, treated with *S. viridosporus* HH1, and non-mycorrhizal, PSM: infected, treated with *S. viridosporus* HH1, and mycorrhizal. Columns superscripted with the same letter(s) are not significantly different according to Tukey’s HSD test at *p* ≤ 0.05. Values represent the mean of five samples per treatment. Error bars represent standard errors.

The effect of the application of *S. viridosporus* HH1 and/or colonization with *R. irregularis* on the disease severity of pea plants infected with Fusarium wilt at 40 dpp is illustrated in Figure 3. The results from the greenhouse experiment showed that no disease symptoms were recorded for the uninfected pea plants, while all infected plants exhibited typical symptoms of Fusarium wilt. In this regard, the highest disease severity was recorded for the infected non-mycorrhizal and untreated pea plants, compared with the uninfected non-mycorrhizal control plants. Treating with *S. viridosporus* HH1 and/or colonization with *R. irregularis* significantly reduced the disease severity of pea plants compared with the untreated, infected, and non-mycorrhizal plants. No significant difference was observed between the disease severity of pea plants which were singly treated with *S. viridosporus* HH1 or colonized with *R. irregularis*. Furthermore, the pea plants treated with the dual treatment showed a lower disease severity value than that of the untreated, infected, and mycorrhizal pea plants. However, the pea plants treated with *S. viridosporus* HH1 and/or colonized with *R. irregularis* recorded a disease severity lower than that of the infected plants that were only treated with the chemical fungicide. No significant difference was recorded between the disease severity in pea plants treated with the dual treatment (77% reduction) and the mycorrhizal plants treated with the chemical fungicide (80% reduction).

### 3.7. Total Phenolics, Activity of Antioxidant Enzymes, Cell Electrolyte Leakage, and TSS

The impacts of treating with the *S. viridosporus* HH1 and/or colonization with *R. irregularis* on the phenolic content, activity of the POD and PPO (30 dpp), cell electrolyte leakage, and the TSS (50 dpp) of pea plants infected with Fusarium wilt are presented in Table 5. The results showed that the infection of pea plants with Fusarium wilt led to an increment in the phenolic content, POD and PPO activity, and cell electrolyte leakage, while the TSS was reduced due to the infection when compared to the uninfected, non-mycorrhizal control plants. At the same time, treating the mycorrhizal colonization with AMF and/or treating with the seaweed extract significantly induced the enzyme activity of the POD and PPO and enhanced the phenolic content and TSS in pea roots when compared to the untreated non-mycorrhizal plants. In comparison, no significant difference was observed for these treatments regarding the electrolyte leakage when compared to the untreated non-mycorrhizal plants. In addition, the infection of pea plants with Fusarium wilt led to a considerable increase in the phenolic content, the activity of POD and PPO enzymes, electrolyte leakage, as well as a decrease in the TSS when compared to the untreated, uninfected non-mycorrhizal plants. Regardless of whether they were infected or not, all tested treatments significantly increased the phenolic content, activity of the POD and PPO, and the TSS content in pea plants, at varying extents, compared with the untreated, uninfected non-mycorrhizal plants. In this regard, the highest values were recorded for the infected pea plants treated with the dual treatment. In contrast, all applied treatments reduced the cell electrolyte leakage in the infected pea plants when compared to the infected non-mycorrhizal plants. No significant difference was observed in the infected pea plants, which were treated only with the chemical fungicide, with regard to the phenolic content and activity of the POD and PPO, when compared with the infected, untreated non-mycorrhizal plants. At the same time, a considerable reduction in the electrolyte leakage and an increment in the TSS content were recorded in the infected plants treated with the fungicide.

### 3.8. Photosynthetic Pigments

The mean photosynthetic pigments in the leaves of infected pea plants in response to the application of *S. viridosporus* HH1 and/or colonization with *R. irregularis* at 40 dpp are presented in Table 6. The infection of pea plants with Fusarium wilt significantly reduced the contents of chl. *a*, chl. *b*, and carotenoids in their leaves, compared with the uninfected non-mycorrhizal plants. All tested treatments enhanced these pigments, to varying extents, compared with the infected non-mycorrhizal plants. The infected pea plants colonized with *R. irregularis* showed total pigments higher than those treated with *S. viridosporus* HH1. However, the highest values of these pigments were recorded for the pea plants treated with the dual treatment. Treating infected pea plants with the chemical fungicide increased the total photosynthetic pigments in their leaves.

### 3.9. Mycorrhizal Colonization Level

**Figure 4:**
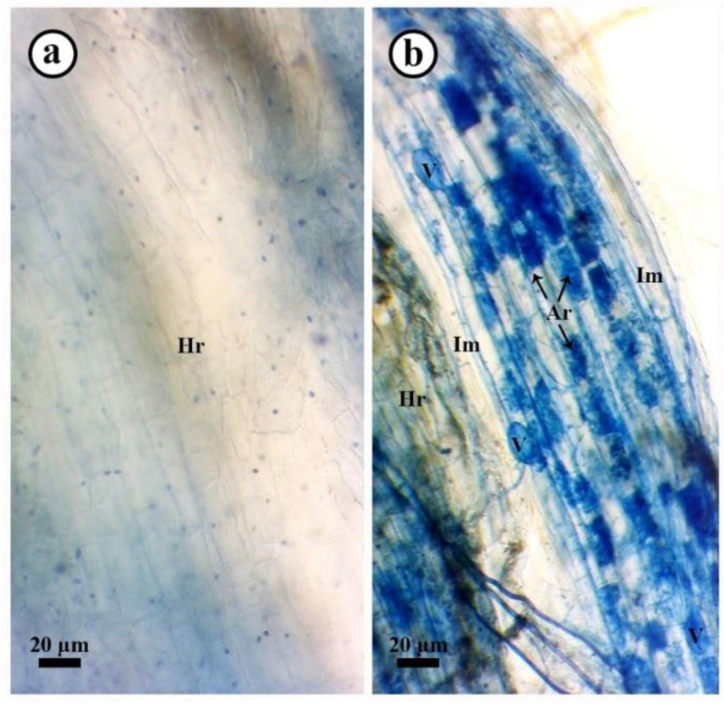
Typical mycorrhizal structures in pea roots colonized with *R. irregularis*. Where (**a**) non-mycorrhizal root (bar = 20 μm), and (**b**) mycorrhizal roots (bar = 20 μm). Where Hr: host root, Im: interaradical mycelia, V: vesicle, and Ar: arbuscule.

The effect of the application of *S. viridosporus* HH1 on the colonization level with *R. irregularis* in the roots of pea plants infected/or not with Fusarium wilt at 30 dpp is shown in Table 7. No mycorrhizal colonization was observed in pea plants not inoculated with *R. irregularis*. In contrast, all treatments inoculated with the *R. irregularis* inoculum showed varied levels of mycorrhizal colonization. A microscopic examination of mycorrhizal pea roots showed typical mycorrhizal structures, which were not observed in the non-mycorrhizal ones (Figure 4). The results obtained revealed that infection with Fusarium wilt, or treated with the chemical fungicide, significantly reduced the mycorrhization level in pea roots when compared to the uninfected mycorrhizal ones. In contrast, treating pea plants with *S. viridosporus* HH1 highly induced the colonization level with *R. irregularis* in their roots when compared to the uninfected, untreated mycorrhizal ones. The highest colonization levels were recorded for pea plants treated with *S. viridosporus* HH1, recording an 85.3% colonization frequency, 44.3% colonization intensity, and 24.4% frequency of arbuscules.

### 3.10. TEM Observations

**Figure 5:**
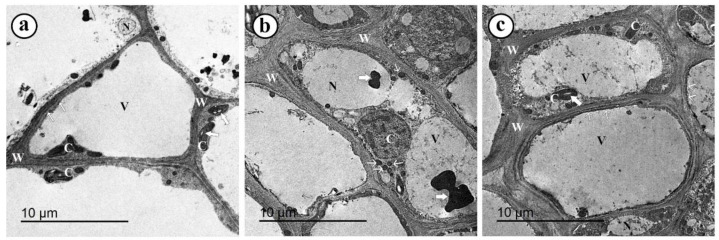
Where (**a**) control plant (uninfected, untreated, and non-mycorrhizal) shows a well-organized and normal cell with a thin cell wall (W) and plasma membranes (short arrows), small nucleus (N), big vacuole (V), and normal chloroplasts (C) containing starch granules (wide arrows) (bar = 10 μm); (**b**) infected plant (untreated, and non-mycorrhizal) showing a disorganized cell with a thick cell wall (W), abnormal chloroplast (C), very electron-dense particles (arrowheads), abnormal vacuole (V), and enlarged nucleus (N), and cytoplasmic granulation (bar = 10 μm); (**c**) mycorrhizal pea plant, infected with Fusarium wilt and treated with *S. viridosporus* HH1 showing a well-organized cell enclosed with a very thick cell wall (W) and cell membrane (arrowheads) and contains a normal nucleus (N), normal chloroplast (C) with starch granules (wide arrows), big vacuole (V), and granulated cytoplasm (bar = 10 μm).

The TEM observations of the hypocotyl region of the control pea plants (uninfected, untreated, and non-mycorrhizal) exhibited well-organized and normal cells with thin cell walls and plasma membranes. The cells comprised small nuclei, big vacuoles, and a set of normal chloroplasts that contained starch granules (Figure 5a). In contrast, TEM observations of infected pea plants (untreated and non-mycorrhizal) showed disorganized cells with various ultrastructural alterations, including cytoplasmic granulation, abnormal chloroplast, very electron-dense particles, thickening of cell walls, abnormal vacuoles, and enlarged nuclei (Figure 5b). Mycorrhizal pea plants (infected and treated with *S. viridosporus* HH1 showed well-organized cells enclosed with very thick cell walls and cell membranes and contained small nuclei, normal chloroplasts, granulated cytoplasm, and large vacuoles (Figure 5c).

## 4. Discussion

Fusarium wilt is a detrimental disease for pea crops, resulting in severe damage to its yield. Mycorrhization of pea plants may provide an effective and eco-friendly alternative to the harmful chemical fungicides used for controlling plant diseases. However, developing synergistically enhanced bioagents for disease management and growth promotion is pivotal for food safety, security, and sustainability. The results obtained showed a potent antifungal activity of *S. viridosporus* HH1 against *F. oxysporum* f.sp. *pisi* in vitro. This result is in agreement with that obtained by Qi et al. [44] on *Streptomyces* sp. SCA3-4, which showed antifungal activity against *F. oxysporum* f.sp. *cubense.* A diverse set of bioactive metabolites, which exhibited antifungal activity, have been reported to be produced by *Streptomyces* spp., such as fatty acids (myristic acid, palmitic acid, stearic acid, oleic acid), (Z)-13-docosenamide, 2,4-Bis(1,1-dimethylethy)-phenol,2,6-Lutidine 3,5-dichloro-4-dodecylthio, and 2,5-Piperazinedione,3,6-bis(2-methylpropyl) [9]. The antifungal mode of action may interfere with the cell membrane permeability, destroy cell structures, inhibit metabolic functions (enzymes), produce siderophores, and/or suppress nucleic acid functions [45]. The results obtained in this study revealed the production of different bioactive substances with antifungal activity, including thioglycolic acid and phthalic acid. In this regard, a potent inhibitory effect was reported for thioglycolic acid against sclerotium formation by the phytopathogen *Verticillium dahliae* that attacks cotton stems [46] and for phthalic acid [47]. The observed antifungal activity of *S. viridosporus* HH1 can be attributed to these bioactive compounds.

Data obtained in this study revealed a synergistic controlling effect against Fusarium wilt in pea plants when treated with *S. viridosporus* HH1 and colonized with *R. irregularis.* The biocontrol of fungal plant diseases using *Streptomyces* spp. Has been widely studied in the last decades [12,48]. Different direct and indirect biocontrol mechanisms have been reported to be utilized by *Streptomyces* spp. [13]. The direct mechanisms against soil microorganisms and pathogens include competition for space or nutrients, especially due to their exploratory growth behavior, antibiosis (antibiotics, enzymes, and volatile compounds), and/or hyperparasitism. In contrast, indirect mechanisms include triggering the plant’s immunity against the pathogen and/or inducing activity of the other soil antagonists against the pathogen [17,18]. In the dual culture test, the antibiosis mechanism seems most likely to be exerted by *S. viridosporus* HH1 than by competition or hyperparasitism. In addition, triggering plant immunity was also confirmed. Resistance priming in various crops using arbuscular mycorrhizal fungi has also been extensively studied against different pathogenic fungi [28,29]. Numerous plant defense responses have been reported to be triggered due to mycorrhizal colonization, including the lignification (thickening) of cell walls, the production of fungitoxic polyphenolic substances, induction of pathogenesis-related proteins, and overexpression of many defense-related genes [27].

The results from this study indicated a synergistic overexpressing effect when applying *S. viridosporus* HH1 and colonization with *R. irregularis* on the transcription factor *JERF3* and the defense-related genes *GLU* and *PR1* in pea plants infected with Fusarium wilt disease. *JERF3* is a transcription factor that regulates a set of defense-related genes through the two signaling pathways, jasmonate and ethylene, triggering the plant’s immunity against different biotic and abiotic stresses [27]. In this study, triggering *JERF3* expression was associated with the upregulation of two defense-related genes, *GLU* and *PR1*, which may explain the recorded induction in pea resistance to Fusarium wilt disease. *GLU* is an antifungal gene that encodes the hydrolyzing enzyme *β*-1,3-glucanase, which catalyzes the degradation of the *β*-1,3/1,6-glucosidic bonds in the glucan molecule—the main structural polysaccharide block of the fungal cell wall [30]. Destroying the cell wall of the invading pathogenic fungus represents an important antifungal mechanism, rendering the fungal cell highly susceptible to full lysis. *PR1* is a pathogenesis-related (PR) gene, which is involved in the plant defense responses to various fungal pathogens [31]. The upregulation of these defense-related genes may explain the reduction in the disease severity reported in this study. Varied inducing effects were observed in this study for the tested single, dual, or triple treatments on the studied genes. Different plant responses were activated in response to the interaction with microorganisms via various signaling pathways, such as abscisic acid (ABA), salicylic acid (SA), JA, and ET. However, crosslinking between these pathways may have occurred [27]. In general, the trophic nature of the microbe determines which signaling pathway will be triggered in the plant. Biotrophs induce the defense responses via the SA pathway, while the necrotrophs elicit the JA pathway. However, plant responses to multiple microbes may differ from that due to individual microbes. In other words, the application of multiple microbes leads to more complex plant responses, which are mediated by synergistic and/or antagonistic signaling pathways [49]. Mycorrhizal colonization has been reported to activate the JA pathway, while plant growth-promoting bacteria, such as *Streptomyces* spp., activate the SA, JA, and/or ET pathways. The observed variation in the inducing effect(s) of the single, dual, or triple treatments on the applied microorganisms in this study on the expression of the studied genes may be discussed in light of the crosslinking between different pathways induced by these microorganisms. Moreover, elevated levels of the fungitoxic phenolic compounds observed in the infected pea plants in response to the dual treatment represent another induced defense response against the fungal infection, restricting the fungal development and growth [29].

One of the most interesting results obtained in this study is the enhancing effect on the activity of the antioxidant enzymes POD and PPO in the infected pea plants in response to their treatment with *S. viridosporus* HH1 and colonization with *R. irregularis*. These enzymes are involved in the antioxidation function scavenging the produced reactive oxygen species (ROS) due to various biotic and abiotic stresses; their activation in a plant is associated with its resistance property [50]. The generated oxidative stress due to infection leads to the excess production of ROS, which causes the destruction of the plant cell organelles and components, membrane oxidation, and its loss of function, and ultimately cell death. Antioxidant enzymes represent the first line of defense against the produced free radicals [51]. These enzymes are normally produced by the plant as a typical defense against any oxidative stress due to different biotic or abiotic stresses; this can be discussed in the context of the observed elevation in the enzyme activity due to the pathogen in this study. However, the enzyme type and activity differ from resistant cultivars to susceptible ones. Eliciting antioxidant enzyme activity improves plant resistance, reducing the damage resultant due to the infection by scavenging ROS via two pivotal roles: catabolism of H_2_O_2_ as well as redox homeostasis. The reported activation of this defense response against the oxidative stress on the infected pea plants, in response to their treatment with *S. viridosporus* HH1 and colonization with *R. irregularis*, was reinforced by the observed decrease in electrolyte leakage. All of these reported modes of action synergistically contributed to the immunity-inducing effect of the applied dual treatment. The TEM observations in this study revealed an enhanced thickening in the infected plant cell wall due to applying *S. viridosporus* HH1 and colonization with *R. irregularis.* This result is in accordance with that obtained with the findings obtained by Rashad et al. [29], who reported an induced cell wall lignification and overexpression in the related genes in sunflower plants as a response to their mycorrhizal colonization with *R. irregularis.* In addition, an increment in the cytoplasmic granulation was also observed from the ultrastructural investigation. This result is in agreement with that obtained by Abdel-Fattah et al. [26], who reported cytoplasmic granulation as a defense response in mycorrhizal common bean plants against infection with Rhizoctonia root rot. Cytoplasmic granulation is a typical hypersensitive response and represents an intermediate stage with vacuolization and condensation in the programmed cell death, restricting the pathogen invasion inside the plant tissue [52,53]. Dynamic changes of the vacuole, particularly vacuolar collapsing, are important in inducing the programmed cell death during the innate immunity against pathogen invasion [54]. In addition to the triggered defense-related responses due to mycorrhizal colonization, the GC-MS analysis from this study indicated that the major secondary metabolites produced by *S. viridosporus* HH1 included 2,3-butanediol and its stereoisomer, constituting around 26% of the total produced metabolites. This volatile organic compound has been reported as an inducer of plant systemic resistance via the ethylene signaling pathway, particularly through ROS homeostasis and overexpressing PR genes [55]. In this regard, Yi et al. [56] reported a considerable upregulation of *GLU* expression in pepper roots when treated with 2,3-butanediol before its infection with *Ralstonia solanacearum.* Moreover, the overexpression of the PR genes, phenylalanine ammonia-lyase (*PAL*) and systemic acquired resistance 8.2 (*SAR8.2*), were also observed. In another study, Park et al. [55] treated tobacco leaves with 2,3-butandiol before their infection with *Phytophtora parasitica* var. *nicotianae.* They noted significant upregulations in the expression of *PR-3*, *PR-4b*, *PR 5 (TLP), PR 5 (OSM), PR 6*, N*PR1* and *PR-1a* when compared to the untreated infected leaves. The immunity-inducing effect observed in this study may be attributed to this bioactive metabolite produced by *S. viridosporus* HH1.

One of the most important results reported in this study is the synergistic growth-promoting effect due to treating pea plants with *S. viridosporus* HH1 and colonization with *R. irregularis*. Mycorrhization of different plant species has been reported to improve their development and yield using multiple modes of action [23,57]. Enhancing water and nutrient acquisition from the soil is one of the most important benefits provided by the mycorrhizal fungi throughout their mutualistic relationship with the hosts. This can be done by extending the fungal extraradical hyphae in the soil to distances deeper than the host roots can do [58]. Improving the phyto-availability of different nutrients in the soil via the secretion of organic acids and hydrolytic enzymes—mainly acid phosphatase—is another discussed growth-promoting mechanism [59]. The induction of photosynthesis performance, photosynthetic pigments, and metabolic enzymes due to mycorrhizal colonization have also been reported [60]. Moreover, various phytohormones have been reported to be released by *R. irregularis,* such as cytokinins, auxins, and gibberellins, which play crucial roles in improving plant growth development, metabolism, and production [61]. In accordance with these reports, we observed elevated levels of the photosynthetic pigments in mycorrhizal pea plants in this study, which may explain the obtained growth-promoting effect of their mycorrhizal colonization. Plant growth promotion, using members of the genus *Streptomyces*, has been investigated on a diverse set of plant species [62]. Different direct and indirect mechanisms have been discussed in this context [18]. Direct plant growth-promoting mechanisms include the secretion of various phytohormones, such as indole-3-acetic acid [63], the production of siderophores [64], nitrogen fixation [65], and reducing plant stress due to ethylene accumulation via the production of 1-aminocyclopropane-1-carboxylate deaminase, which leads to an enhancement of plant tolerance to abiotic stresses [66]. In addition, enhancement of the photosynthetic pigments content and efficiency of the photosynthesis apparatus, due to the induction of their enzymatic performance in response to the application of *Streptomyces* spp., was also reported [67]. Enhancement of the photosynthetic pigment contents of pea crops, which was observed in this study due to the application of *S. viridosporus* HH1, is in agreement with the results obtained by Passari et al. [68], who reported an increment in the content of chlorophyll *a* and *b* as well as the photochemical quantum yield and electron transport rate of photosystem II in tomato plants inoculated with *S. thermocarboxydus* BPSAC147. Enhancing the photosynthetic pigments and the photosynthetic apparatus performance leads to improving plant growth via glucose synthesis. In addition to their exploration growth, *Streptomyces* spp. can indirectly promote plant growth by producing volatile organic substances that act as gene regulators and elicitors, modulating the soil microbiome responses, diversity, and dynamics [69]. The bioactive compound 2,3-butanediol produced by *S. viridosporus* HH1 has been reported as a plant growth promoter. Wu et al. [70] reported a 1.88-fold increment in the fresh weight of *Arabidopsis thaliana* plantlets treated with 2,3-butanediol (500 μg).

## 5. Conclusions

In this study, the obtained results indicated the antifungal activity of *S. viridosporus* HH1 against *F. oxysporum* f.sp. *pisi* in vitro. The GC-MS analysis revealed the production of different bioactive compounds by *S. viridosporus* HH1, including 2,3-butanediol, thioglycolic acid, and phthalic acid. Treatment with *S. viridosporus* HH1 and colonization with *R. irregularis* of pea plants infected with Fusarium wilt showed a synergistic biocontrol activity, resulting in a 77% reduction in disease severity. In addition, this dual treatment upregulated the transcription factor *JERF3* and the defense-related genes (*GLU* and *PR1*), enhanced the total phenolic content, induced the antioxidant activity of the POD and PPO in pea plants, reduced the antioxidant stress, and improved their hypersensitivity at the ultrastructural level in response to the Fusarium wilt pathogen. Moreover, a synergistic growth-promoting effect was also recorded in pea plants in response to this dual treatment. In this regard, elevated levels of photosynthetic pigments were observed in pea leaves due to this dual treatment. Moreover, an improvement in the evaluated growth and yield parameters was also observed, and when we applied *S. viridosporus*, it enhanced the colonization level with *R. irregularis* in pea roots. Based on the obtained data, we can conclude that treating pea plants with *S. viridosporus* HH1 and colonization with *R. irregularis* have synergistic biocontrol activity and growth-promoting effects on pea plants against Fusarium wilt. Despite its eco-safety and effectiveness, a field evaluation of this treatment before a recommendation is quite necessary.

## Figures and Tables

**Table 1:** Primer sequences used in the molecular investigation.

Gene Description	Abbrev.	Accession No.	Sequence (5′-3′)
Jasmonate and ethylene-responsive factor 3	*JERF3*-F *JERF3*-R	AY383630	GCCATTTGCCTTCTCTGCTTC GCAGCAGCATCCTTGTCTGA
*β*-1,3-glucanase	*GLU*-F *GLU*-R	M80604	TTTCGATGCCCTTGTGGATT CGGCCAACCACTTTCCGATAC
Pathogenesis-related protein 1	*PR1*-F *PR1*-R	M69247	ACTTGGCATCCCGAGCACAA CTCGGACACCCACAATTGCA
Elongation factor 1-α	*EF1-α*-F *EF1-α*-R	EC959059	GAACTGGGTGCTTGATAGGC AACCAAAATATCCGGAGTAAAAGA

**Table 2:** Secondary metabolites produced by *S. viridosporus* HH1 identified using GC-MS system.

Peak #	Retention Time (min)	Peak Area (%)	Compound Name
1	1.592	13.35	Thioglycolic acid
2	3.139	17.26	2,3-Butanediol
3	3.266	8.90	d-2,3-Butanediol
4	3.647	9.21	2-Ethoxypropane
5	5.003	0.09	1-Methoxy-2-butanol
6	5.891	0.05	Acetal
7	6.895	0.05	Isopropyl isobutyrate
8	17.558	4.88	Cyclohexasiloxane, dodecamethyl
9	22.661	11.74	Cycloheptasiloxane, tetradecamethyl-
10	24.470	0.32	Isopropoxy-3,3,3-trimethyl-[(trimethylsilyl)oxy]
11	26.910	6.65	Hexasiloxane, tetradecamethyl-
12	27.424	0.26	Homogentisic acid, bis(tert-butyldimethylsilyl)
13	28.520	0.11	Tetradecamethylhexasiloxane
14	29.643	13.78	Phthalic acid
15	30.535	1.73	Hexadecamethylheptasiloxane
16	31.900	6.94	Phloroglucinol, tris(trimethylsilyl ether)
17	33.761	0.55	Glaucine
18	34.659	1.52	Phenyltrimethylsilane
19	40.785	2.33	1,2-Diphenyltetramethyldisilane
20	42.271	0.27	Pyrrolidino[1,2-a]piperazine-3,6-dione

**Table 3:** Effects of application of *S. viridosporus* HH1 and/or colonization with *R. irregularis* on growth parameters of pea plants (cv. Master-B) infected with Fusarium wilt at 50 days post planting ^a^.

Treatment	Mycorrhizal Status	Shoot Height (cm)	Root Length (cm)	Shoot Dry Weight (g)	Root Dry Weight (g)	Number of Leaves/Plant	Leaf Area (cm^2^)
C	NM	46.0 ± 2.6	5.7 ± 0.7	1.7 ± 0.3	0.13 ± 0.04	5.3 ± 0.4	38.7 ± 3.0
	M	58.7 ± 3.1	16.1 ± 0.9	2.6 ± 0.5	0.18 ± 0.03	7.7 ± 0.5	52.4 ± 2.7
P	NM	36.1 ± 1.9	4.2 ± 0.6	1.0 ± 0.2	0.10 ± 0.04	4.0 ± 0.5	34.0 ± 2.3
	M	54.3 ± 2.4	12.3 ± 0.4	2.4 ± 0.3	0.15 ± 0.03	8.0 ± 0.4	44.3 ± 2.8
S	NM	55.5 ± 2.7	12.0 ± 0.5	2.6 ± 0.2	0.16 ± 0.02	7.3 ± 0.6	51.5 ± 3.1
	M	62.7 ± 2.5	18.5 ± 0.9	2.8 ± 0.6	0.21 ± 0.04	7.3 ± 0.6	61.4 ± 3.6
PS	NM	51.2 ± 1.9	10.3 ± 0.7	1.6 ± 0.2	0.13 ± 0.02	6.7 ± 0.9	51.1 ± 2.9
	M	58.8 ± 2.1	16.3 ± 0.6	2.4 ± 0.3	0.19 ± 0.03	6.7 ± 0.3	55.1 ± 3.2
PF	NM	44.8 ± 2.0	5.7 ± 0.3	1.7 ± 0.2	0.13 ± 0.04	5.3 ± 0.3	35.5 ± 2.5
	M	49.5 ± 1.8	7.3 ± 0.6	2.1 ± 0.4	0.14 ± 0.05	7.3 ± 0.4	36.4 ± 2.0
LSD (*p* < 0.05)		3.5	1.5	0.7	0.03	1.28	4.4
Treatment		***	***	**	*	*	***
Mycorrhiza		**	**	***	**	*	***
Treatment × Mycorrhiza		***	**	**	*	*	***

^a^ C: uninfected and untreated control, P: infected and untreated, S: uninfected and treated with *S. viridosporus* HH1, PS: infected and treated with *S. viridosporus* HH1, PF: infected and treated with fungicide (Tendro 40% FS), NM: non-mycorrhizal, M: mycorrhizal. * Significant at *p* < 0.05, ** significant at *p* < 0.01, and *** significant at *p* < 0.001.

**Table 4:** Effects of application of *S. viridosporus* HH1 and/or colonization with *R. irregularis* on yield parameters of pea plants (cv. Master-B) infected with Fusarium wilt at 70 days post planting ^a^.

Treatment	Mycorrhizal Status	No. Of Pods/Plant	Pod Weight (g)	Pod Length (cm)	Pod Width (cm)	Yield/Plant (g)	No. of Seeds/Pod
C	NM	2.0 ± 0.6	4.6 ± 0.8	5.4 ± 0.3	1.5 ± 0.05	9.1 ± 0.8	4.7 ± 0.6
	M	3.3 ± 0.3	5.3 ± 0.9	8.0 ± 0.6	1.6 ± 0.04	17.2 ± 1.1	7.0 ± 0.8
P	NM	2.0 ± 0.3	2.3 ± 0.3	4.6 ± 0.3	1.17 ± 0.05	4.6 ± 0.6	3.0 ± 0.5
	M	3.0 ± 0.4	4.9 ± 0.4	7.3 ± 0.3	1.16 ± 0.04	14.9 ± 1.3	6.7 ± 0.7
S	NM	3.3 ± 0.3	4.9 ± 0.5	7.6 ± 0.6	1.53 ± 0.03	16.4 ± 1.8	4.4 ± 0.3
	M	3.7 ± 0.6	5.5 ± 0.6	9.5 ± 0.2	1.63 ± 0.03	19.4 ± 2.0	5.1 ± 0.5
PS	NM	3.0 ± 0.3	4.7 ± 0.2	7.3 ± 0.2	1.47 ± 0.04	14.0 ± 0.5	6.7 ± 0.3
	M	3.7 ± 0.9	5.0 ± 0.7	9.0 ± 0.3	1.63 ± 0.02	16.6 ± 0.9	6.0 ± 0.6
PF	NM	2.0 ± 0.3	3.8 ± 0.6	5.6 ± 0.4	1.47 ± 0.02	8.8 ± 0.6	4.0 ± 0.3
	M	3.0 ± 0.2	4.0 ± 0.4	6.9 ± 0.3	1.53 ± 0.03	10.8 ± 0.8	4.4 ± 0.2
LSD (*p* < 0.05)		0.98	1.5	0.8	0.2	4.1	1.4
Treatment		**	*	***	Ns	**	*
Mycorrhiza		**	*	*	Ns	**	*
Treatment × Mycorrhiza		*	*	*	Ns	**	*

^a^ C: uninfected and untreated control, P: infected and untreated, S: uninfected and treated with *S. viridosporus* HH1, PS: infected and treated with *S. viridosporus* HH1, PF: infected and treated with fungicide (Tendro 40% FS), NM: non-mycorrhizal, M: mycorrhizal. Ns: not significant, * Significant at *p* < 0.05, ** significant at *p* < 0.01, and *** significant at *p* < 0.001.

**Table 5:** Effects of application of *S. viridosporus* HH1 and/or colonization with *R. irregularis* on phenolic content, activity of antioxidant enzymes (30 days post planting), electrolyte leakage, and total soluble solids (50 days post planting) of pea plants (cv. Master-B) infected with Fusarium wilt ^a^.

Treatment	Mycorrhizal Status	Phenolic Content (mg g^−1^ fwt)	Peroxidase (∆A_470_ min^−1^ g^−1^ fwt)	Polyphenol Oxidase (∆A_420_ min^−1^ g^−1^ fwt)	Electrolyte Leakage (%)	Soluble Solids Content (°Brix)
C	NM	394.6 ± 8.5	0.9 ± 0.08	1.0 ± 0.04	55.3 ± 1.02	14.1 ± 0.7
	M	570.2 ± 7.4	1.8 ± 0.05	1.5 ± 0.03	55.9 ± 1.1	16.2 ± 0.5
P	NM	568.9 ± 5.6	1.4 ± 0.03	1.3 ± 0.04	118.4 ± 1.7	8.1 ± 1.0
	M	571.0 ± 6.8	2.3 ± 0.09	1.9 ± 0.06	84.5 ± 1.3	15.9 ± 0.6
S	NM	500.2 ± 9.2	1.7 ± 0.04	1.5 ± 0.04	56.9 ± 1.2	15.0 ± 0.4
	M	604.4 ± 10.1	2.0 ± 0.05	1.4 ± 0.04	55.4 ± 1.4	17.3 ± 0.3
PS	NM	763.1 ± 12.3	2.0 ± 0.03	1.6 ± 0.03	84.9 ± 1.3	14.8 ± 0.4
	M	1135.2 ± 20.4	2.3 ± 0.05	1.9 ± 0.03	75.4 ± 1.1	16.4 ± 0.2
PF	NM	537.1 ± 6.3	1.3 ± 0.09	1.3 ± 0.07	83.1 ± 1.5	12.3 ± 0.8
	M	667.7 ± 8.4	1.9 ± 0.04	1.8 ± 0.08	84.5 ± 1.4	12.9 ± 0.6
LSD (*p* < 0.05)		163.1	0.2	0.2	3.5	1.7
Treatment		***	***	***	***	***
Mycorrhiza		**	**	***	*	**
Treatment × Mycorrhiza		*	***	***	***	***

^a^ C: uninfected and untreated control, P: infected and untreated, S: uninfected and treated with *S. viridosporus* HH1, PS: infected and treated with *S. viridosporus* HH1, PF: infected and treated with fungicide (Tendro 40% FS), NM: non-mycorrhizal, M: mycorrhizal. * Significant at *p* < 0.05, ** significant at *p* < 0.01, and *** significant at *p* < 0.001.

**Table 6:** Effects of application of *S. viridosporus* HH1 and/or colonization with *R. irregularis* on total photosynthetic pigments (40 days post planting) in leaves of pea plants (cv. Master-B) infected with Fusarium wilt ^a^.

Treatment	Mycorrhizal Status	Chl. *a* (mg g^−1^ fwt)	Chl. *b* (mg g^−1^ fwt)	Carotenoids (mg g^−1^ fwt)	Total Pigments (mg g^−1^ fwt)
C	NM	2.5 ± 0.5	0.8 ± 0.06	0.4 ± 0.04	3.7 ± 0.9
	M	3.0 ± 0.4	0.7 ± 0.05	0.8 ± 0.05	4.6 ± 0.4
P	NM	0.6 ± 0.1	0.1 ± 0.02	0.1 ± 0.01	0.9 ± 0.09
	M	2.4 ± 0.6	0.5 ± 0.04	0.6 ± 0.02	3.6 ± 0.5
S	NM	2.7 ± 0.3	1.0 ± 0.02	0.6 ± 0.02	4.3 ± 0.3
	M	3.8 ± 0.6	1.2 ± 0.07	0.7 ± 0.06	5.7 ± 0.6
PS	NM	2.2 ± 0.4	0.4 ± 0.03	0.5 ± 0.04	3.1 ± 0.4
	M	2.7 ± 0.2	1.2 ± 0.05	0.3 ± 0.05	4.2 ± 0.3
PF	NM	2.1 ± 0.5	0.5 ± 0.01	0.4 ± 0.03	2.9 ± 0.3
	M	2.3 ± 0.4	0.4 ± 0.02	0.6 ± 0.06	3.3 ± 0.4
LSD (*p* < 0.05)		0.2	0.2	0.1	0.3
Treatment		***	**	*	***
Mycorrhiza		***	**	***	***
Treatment × Mycorrhiza		***	*	**	***

^a^ C: uninfected and untreated control, P: infected and untreated, S: uninfected and treated with *S. viridosporus* HH1, PS: infected and treated with *S. viridosporus* HH1, PF: infected and treated with fungicide (Tendro 40% FS), NM: non-mycorrhizal, M: mycorrhizal. * Significant at *p* < 0.05, ** significant at *p* < 0.01, and *** significant at *p* < 0.001.

**Table 7:** Effects of application of *S. viridosporus* HH1 on colonization level with *R. irregularis* in roots of pea plants (cv. Master-B) infected/or not with Fusarium wilt at 30 days post planting ^a^.

Treatment	Mycorrhizal Status	FC (%)	IC (%)	FA (%)
C	NM	0	0	0
	M	78.3 ± 1.2	35.4 ± 1.0	11.6 ± 0.9
P	NM	0	0	0
	M	54.6 ± 0.9	17.2 ± 0.7	8.4 ± 0.5
S	NM	0	0	0
	M	85.3 ± 1.3	44.3 ± 1.1	24.4 ± 1.0
PS	NM	0	0	0
	M	80.5 ± 0.8	40.7 ± 1.1	20.7 ± 0.6
PF	NM	0	0	0
	M	75.4 ± 0.7	24.8 ± 0.8	10.0 ± 0.5
LSD (*p* < 0.05)		2.4	3.3	1.5
Treatment		***	***	***
Mycorrhiza		***	***	***
Treatment × Mycorrhiza		***	**	**

^a^ FC: frequency of colonization, IC: intensity of colonization, FA: frequency of arbuscules, C: uninfected and untreated control, P: infected and untreated, S: uninfected and treated with *S. viridosporus* HH1, PS: infected, and treated with *S. viridosporus* HH1, PF: infected, and treated with fungicide (Tendro 40% FS), NM: non-mycorrhizal, M: mycorrhizal. ** significant at *p* < 0.01, and *** significant at *p* < 0.001.

## Data Availability

Not applicable.
